# Spinal Dural Arteriovenous Fistula: The Missing-Piece Sign

**DOI:** 10.31486/toj.21.0110

**Published:** 2022

**Authors:** Michael Kelley, Daniel April, Bridget Bagert, James Milburn, Andrew Steven

**Affiliations:** ^1^ Department of Radiology, Ochsner Clinic Foundation, New Orleans, LA; ^2^ Department of Neurology, Ochsner Clinic Foundation, New Orleans, LA; ^3^ The University of Queensland Faculty of Medicine, Ochsner Clinical School, New Orleans, LA

## INTRODUCTION

Spinal dural arteriovenous fistulas (sDAVFs) are a rare and often underdiagnosed spinal pathology. They occur predominantly in males in the fifth or sixth decade of life and most commonly involve the thoracolumbar region, although sDAVFs can occur anywhere along the spinal cord.^1^ Presenting clinical symptoms are often insidious in onset and of nonspecific nature, such as lower extremity peripheral neuropathies, pain, and exertional leg weakness. Symptoms may progress slowly, over several years, to severe myelopathy with paraplegia.^2^ These symptoms and the accompanying imaging findings are often attributed to more common and occasional coexistent pathologic processes such as degenerative spinal stenosis or demyelinating/inflammatory myelitis.^2,3^ Early diagnosis of sDAVFs is important; deficits are potentially reversible, but delayed treatment may result in irreversible neurologic disability.^4,5^ Imaging diagnosis primarily relies on magnetic resonance imaging (MRI) and conventional spinal angiography. Once an sDAVF is identified, treatment is either endovascular embolization or surgical ligation of the fistula. We describe a case of sDAVF in a patient who underwent treatment with Onyx (Medtronic) embolization.

## CASE DESCRIPTION

A 76-year-old female with a medical history of hypothyroidism and lumbar spinal stenosis was referred to our neurology clinic by her primary care physician (PCP). She had initially presented to her PCP at an outside hospital 4 years prior to this neurology clinic visit because of sudden onset leg weakness that made walking very difficult and for which she required assistance. She had episodes of collapsing with spontaneous resolution of symptoms after a few days of rest. After the initial presentation to her PCP, she had 3 similar episodes, all spontaneously resolving after 3 to 5 days.

Her PCP prescribed physical therapy intermittently during this 4-year period without significant improvement. Failing conservative management, and 5 months prior to her appointment in our neurology clinic, the patient was treated at an outside institution with transforaminal lumbar interbody fusion at the L4-L5 spinal level for grade 2 spondylolisthesis and lumbar spinal stenosis. She tolerated the procedure well with no complications and recovered at a rehabilitation facility. One month after surgery and following discharge from the rehabilitation facility, the patient began to have left-sided sciatica and radiculopathy with subsequent neurogenic claudication and bilateral lower extremity numbness. She also reported functional deficits of urinary incontinence and increased urinary frequency for which she performed pelvic floor exercises 3 times weekly.

**Figure 1.**
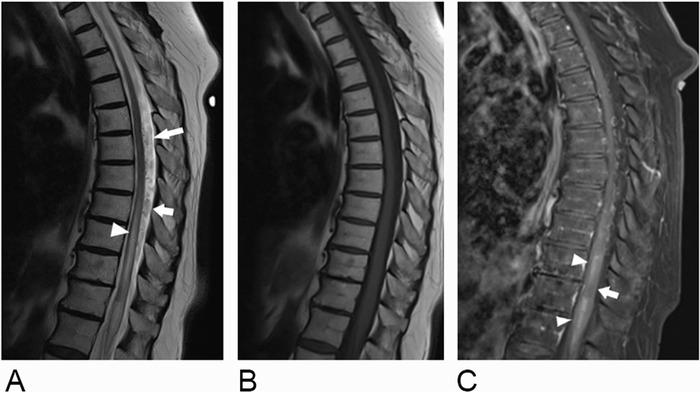
Magnetic resonance imaging of the thoracic spine. (A) Sagittal T2-weighted image through the level of the mid-thoracic spinal cord demonstrates long segment central cord hyperintensity and expansion in the lower thoracic cord (arrowhead) and prominent serpentine T2 flow voids in the posterior intradural space (arrows). Sagittal T1-weighted images (B) before and (C) after contrast administration with fat saturation demonstrate corresponding patchy central cord enhancement (arrowheads) with a discrete nonenhancing segment known as the missing-piece sign (arrow).

**Figure 2.**
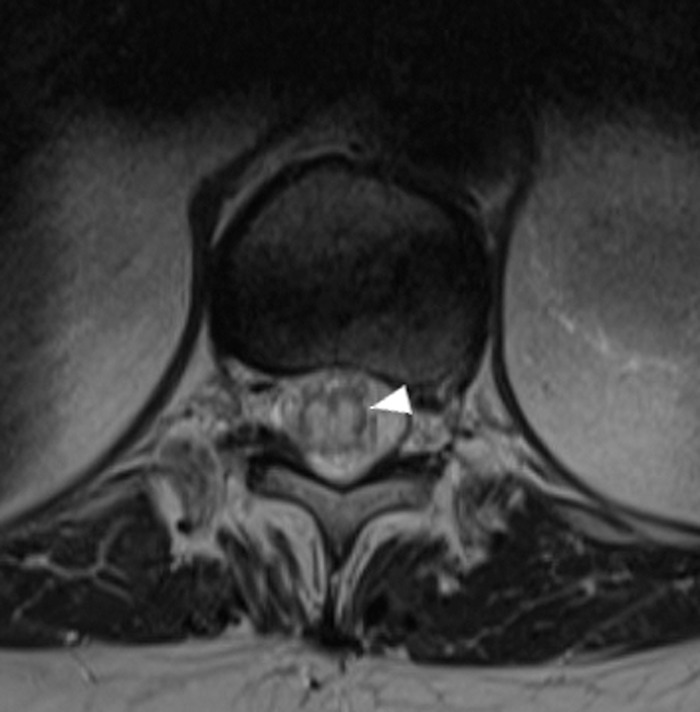
Axial T2-weighted image through the level of the mid-thoracic spinal cord demonstrates long segment central cord hyperintensity and expansion in the lower thoracic cord (arrowhead).

Upon re-presentation with these complaints, her PCP obtained an MRI (Figures 1 and 2) that revealed a long segment of central cord expansion and hyperintensity extending from the thoracic region through the tip of the conus with prominent vascular flow voids in the posterior intradural extramedullary space. These findings prompted referral to our neurology clinic at which time an initial workup for inflammatory and demyelinating causes included cerebral spinal fluid analysis with multiple sclerosis profile and extensive laboratory workup (myelin oligodendrocyte glycoprotein-immunoglobulin G1, neuromyelitis optica-aquaporin-4-immunoglobulin G, angiotensin-converting enzyme, rheumatoid factor, anti-Sjögren syndrome type B antibodies, and protein and immunofixation electrophoresis). All tests were normal.

**Figure 3.**
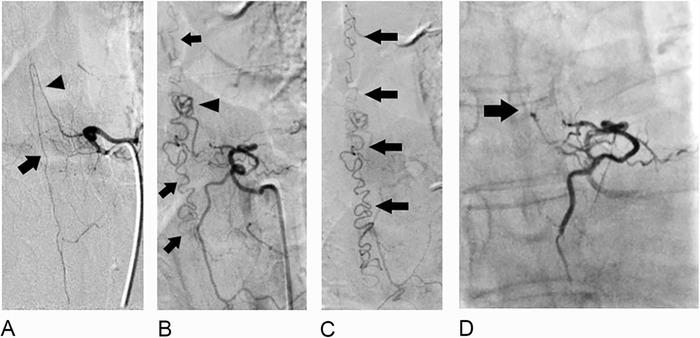
Direct spinal angiography images of the mid-thoracic spine. (A) The artery of Adamkiewicz (arrowhead) arises from the left T6 segmental artery supplying a normal anterior spinal artery (arrow). (B and C) Selective left T8 segmental injection demonstrates an arteriovenous fistula supplied by small branches of the left radicular artery (arrowhead). This arteriovenous fistula drained into the spinal canal via numerous dilated perimedullary veins extending both cranially and caudally (arrows). (D) A Scepter balloon microcatheter (MicroVention Inc) was advanced into the left T8 segmental artery and inflated for protection from reflux. Then 0.6 cc of Onyx-18 (Medtronic) was injected, opacifying the feeding vessels and fistula. After embolization, the Onyx cast is shown with filling of the arterial supply to the fistula (arrow). No supply was visualized from the T7, T8, and T9 segmental arteries after embolization, suggesting complete occlusion.

The patient was referred for conventional angiographic evaluation (Figure 3). An sDAVF was identified arising from the left T6 segmental artery with a dilated perimedullary venous network extending cranially and caudally. Embolization using Onyx (Medtronic) was performed successfully. Following the procedure, the patient was admitted because of subjective complaints of worsening bilateral lower extremity weakness. She remained stable and was discharged after 3 days of hospitalization with outpatient physical therapy.

**Figure 4.**
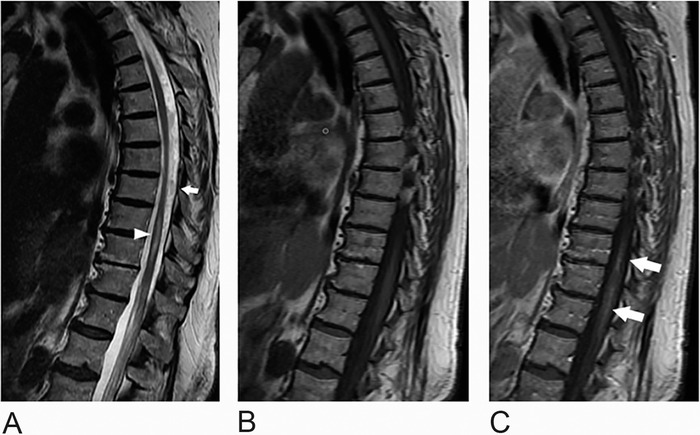
Magnetic resonance imaging of the thoracic spine 14 months postembolization. (A) Sagittal T2-weighted image demonstrates persistent but considerably decreased patchy central cord T2 hyperintensity extending from the T8 level to the conus (arrowhead) with decreased prominence of the venous flow voids in the posterior intradural space (arrow). Sagittal T1-weighted images (B) before and (C) after contrast administration demonstrate mild persistent faint enhancement, considerably improved since the initial examination (arrows).

**Figure 5.**
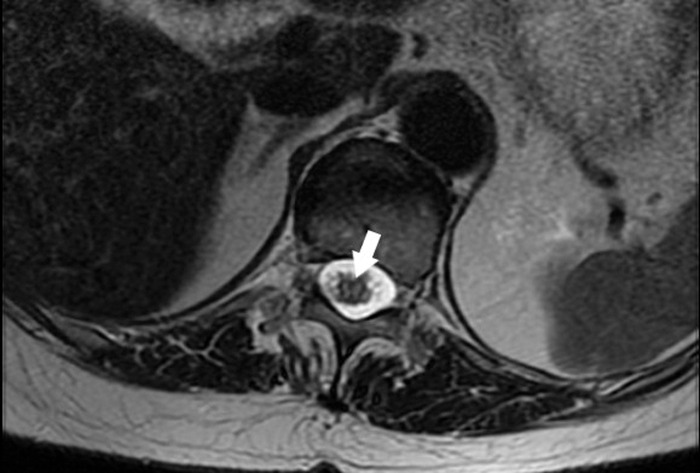
Axial T2-weighted image at the T12 level 14 months postembolization shows mild persistent hyperintense signal with resolved expansion of the central cord (arrow).

At 1-year follow-up, her symptoms were mildly improved. She still required a rolling walker to ambulate and occasionally self-catheterized because of urinary retention. MRI 14 months following sDAVF embolization (Figures 4 and 5) revealed persistent but improved abnormal cord signal and enhancement extending from the T8 level to the conus. Repeat conventional angiogram did not reveal any residual arteriovenous shunting or venous congestion.

## ETIOLOGY

sDAVFs, although a rare pathology, are the most common vascular shunts of the spine, characterized by an abnormal communication between arteries and veins within the dura.^1^ These connections are classically located within the dura mater near spinal nerve roots. Branches of the radiculomeningeal artery make up the majority of the arterial components of the shunt, while the venous component usually consists of a radicular vein.^5-7^ Although their exact etiology is not fully understood, sDAVFs are presumed to be acquired lesions with several potential predisposing factors, including thrombosis of the extradural spinal veins and traumatic injury, although some lesions may be idiopathic.^5,7^ Communicating arteries cause decreased arteriovenous pressure gradients, leading to decreased venous drainage and subsequent venous congestion with intramedullary edema. This congestion can cause chronic hypoxia which, if left untreated, results in ischemia and necrosis.^7^

## RADIOGRAPHIC APPEARANCE AND MANAGEMENT

Initial diagnostic evaluation of sDAVFs may begin with MRI. Findings include swelling/enlargement of the spinal cord with corresponding T2 hyperintensity and T1 hypointensity, predominantly affecting the lower thoracic region and conus and often extending along multiple segments.^3-5,8^ The superior and inferior margins are often described as “flame shaped.” The spinal cord gray and white matter are both affected. Areas of signal change and cord enlargement do not necessarily correspond to the fistula location.^3^ In a case series of 147 patients with spinal MRIs, Muralidharan et al reported that T2 hyperintensity was observed within the conus in 95% of patients.^9^ Principal involvement of the conus is thought to be attributable to fewer venous egress routes compared to higher spinal cord segments.^6,7^

Many pathologic processes can also result in an expanded spinal cord with intramedullary T2 hyperintensity. In a case series of misdiagnosed sDAVFs, the most common misdiagnoses were spinal stenosis (24.5%), myelopathy not otherwise specified (18.9%), and transverse myelitis (17%).^2^ However, a few subtle but characteristic imaging features can aid in the diagnosis. Long-standing venous hypertension and congestion resulting in chronic hypoxia of the spinal cord may induce a characteristic peripheral rim of low T2 signal.^7-9^ Another subtle but highly suggestive finding is the presence of prominent intrathecal vascular flow voids surrounding the lower spinal cord, which represent a dilated perimedullary venous plexus. Identification of these subtle findings is a critical step in accurately diagnosing a vascular malformation, and sensitivity can be improved by the addition of high-resolution 3-dimensional T2-weighted sequences (ie, T2-SPACE.)^10^

A characteristic enhancement pattern for sDAVFs known as the missing-piece sign has been recently (2018) described in the literature.^4^ The missing-piece sign can be seen on MRI and is defined as at least one discrete region of nonenhancement within a long segment of intense spinal cord gadolinium enhancement.^4^ In a case series, 86% of patients had intraparenchymal contrast enhancement, and the missing-piece sign was present in 43% of patients.^4^ This pattern of enhancement appears specific for the diagnosis of sDAVFs when compared to other patterns of spinal cord enhancement. The differential diagnoses for enhancing spinal cord lesions include demyelinating processes, neoplasm, and infarction. Demyelinating lesions of the spinal cord often produce an incomplete ring of enhancement. For infarction, the enhancement pattern depends on age of infarct, and long spinal cord segments are typically involved, with the anterior cord being the most common site. When distinguishing intramedullary tumors, the enhancement pattern is variable and may be heterogenous in astrocytomas, solid in ependymomas, or small compared to the extent of edema in metastases.^11^

A missing-piece sign pattern of enhancement, along with perimedullary vascular flow voids, should prompt additional evaluation with angiography. Angiography is a minimally invasive diagnostic procedure that uses fluoroscopy to visualize catheterized blood vessels with injection of contrast dye. Contrast stasis within radiculomedullary arteries, delayed venous return following injection, and retrograde contrast uptake within radiculomedullary veins are common findings indicating venous congestion and underlying shunting.^7^ Although computed tomography angiography and magnetic resonance angiography have been shown to be useful in identifying, classifying, and aiding in treatment planning of sDAVFs, conventional spinal angiography remains the gold standard for diagnosis and classification.^5,12^ Risks associated with spinal angiography include potential nephrotoxicity from iodinated contrast, increased radiation exposure, and risks associated with the invasive nature of the examination. These risks highlight the importance of recognizing and localizing the fistula on initial imaging to permit selective catheterization of vessels, thereby minimizing examination time and mitigating risks.^5^

General management and treatment strategies depend on many factors, such as clinical symptoms, fistula localization and classification, and risk of hemorrhage. The complexity and variability of sDAVFs warrant a multidisciplinary approach and careful planning.^13,14^

Multiple classification systems for DAVFs have been proposed. A popular classification system for DAVFs is the Cognard classification that categorizes DAVFs into 5 types based upon lesion location, presence of cortical venous drainage, flow direction, and presence of venous ectasia.^13,14^ The Cognard classification has clinical utility in stratifying lesion risks of intracranial hemorrhage and increasingly aggressive clinical course.^13,14^ Type I lesions have antegrade drainage directly into the venous sinus. Type II lesions drain into the dural sinus and are further subdivided into IIA, IIB, or IIA+B based upon antegrade/retrograde flow and presence of cortical venous drainage. Type III lesions drain directly into cortical veins, and Type IV lesions drain into cortical veins with venous ectasia. Type V lesions have spinal perimedullary venous drainage and are associated with edema and progressive myelopathy. Our case is a Cognard V lesion.^13^

Management strategies include conservative management with surveillance imaging, endovascular embolization, and surgical ligation. Conservative management may be appropriate for asymptomatic sDAVFs, but close clinical and imaging surveillance is necessary because of the risk of progression to symptomatic lesions.^15,16^ Imaging surveillance can be performed using MRI, looking for features of developing edema/myelopathy, and clinical surveillance can be conducted by the neurointerventional team.^13-16^ Once lesions are symptomatic, the natural clinical course is gradual worsening with the potential for irreversible spinal cord injury, and early treatment should be considered to alleviate patient symptoms and to mitigate clinical deterioration.^5^ At symptom onset, initial neurologic status has been shown to significantly influence clinical outcomes.^5^ Not classically associated with hemorrhage compared with more aggressive Cognard lesions, sDAVFs have an overall rate of bleeding estimated at 1.8%; however, if an initial hemorrhage is present, the risk of rebleeding within the first 2 weeks has been estimated to be as high as 35%.^15,17^ Treatment options for sDAVFs include endovascular embolization, surgical ligation, or a combination of both, with treatment planning best suited for discussion among multidisciplinary teams.^13-16^

## CONCLUSION

Because of the insidious onset and nonspecific nature of presenting clinical symptoms, sDAVFs are frequently overlooked and often misdiagnosed. A unique pattern of enhancement has been described that appears fairly specific for this diagnosis. Familiarity with imaging features of sDAVFs can prevent unnecessary delay in diagnosis and treatment, as well as further invasive testing for pathologies with similar presenting symptoms.

## Notes

*This article meets the Accreditation Council for Graduate Medical Education and the American Board of Medical Specialties Maintenance of Certification competencies for Patient Care and Medical Knowledge.*
